# Eosinophilic mucin chronic rhinosinusitis with orbital involvement: management strategies based on clinical presentation

**Published:** 2020-12-31

**Authors:** Archana A Nair, Andrea A Tooley, Fang Zhou, Seth Lieberman, Payal Patel, Irina Belinsky

**Affiliations:** New York University, Department of Ophthalmology,; New York University, Department of Ophthalmology,; 1 New York University, Department of Pathology, New York, USA; 2 New York University, Department of Otolaryngology-Head and Neck Surgery,

**Keywords:** Allergic fungal sinusitis, orbital, subperiosteam abscess

## Abstract

Allergic fungal sinusitis (AFS) and eosinophilic mucin chronic rhinosinusitis (EMCRS) are subtypes of a chronic rhinosinusitis with eosinophilia that have different diagnostic criteria but are phenotypically similar. Ophthalmic complications may be the presenting symptoms. Treatment of ophthalmic complications is typically directed at reducing the inflammatory burden in the sinuses and rarely requires direct surgical intervention. However, atypical cases with associated subperiosteal abscess may necessitate orbital surgery. The authors present 2 cases of EMCRS with orbital involvement – one that responded to the traditional treatment of oral corticosteroids and functional endoscopic sinus surgery (FESS), and the other requiring surgical drainage of a subperiosteal abscess in order to describe the management strategies based on clinical presentation.

Allergic fungal sinusitis (AFS) is a distinct form of chronic rhinosinusitis, diagnosed using Bent-Kuhn criteria: (a) nasal polyposis, (b) radiographic findings, (c) eosinophilic mucus without fungal invasion into sinuses, (d) positive fungal stain of sinus contents, and (e) Type I hypersensitivity by history, serology, or skin testing.^[1]^ Some patients have a similar phenotype to AFS but lack the type I hypersensitivity or positive fungal stains. This entity is referred to as eosinophilic mucinous chronic rhinosinusitis (EMCRS).^[2]^ AFS and EMCRS typically occur in immunocompetent patients, often with a history of atopy and asthma. Characteristic buildup of thick mucin that results from the extensive eosinophilic activation causes compression of the skull base and orbit, resulting in proptosis, ptosis, epiphora, diplopia, and visual loss from optic neuropathy.^[3]^ Studies have suggested that up to 39% of patients have ophthalmic complications.^[4]^ Computed Tomography (CT), the initial study of choice, demonstrates opacification of multiple sinuses, sinus expansion, hyper-dense material corresponding to eosinophilic mucin, and erosion of surrounding bone including the orbit and/or skull base.^[5]^ Direct orbital extension occurs by bony demineralization and erosion. Orbital involvement in these diseases presents clinically similar to orbital cellulitis, which is commonly from sinus disease, but management with sinus surgery and corticosteroids differs. Here, we describe 2 contrasting cases of EMCRS with orbital involvement, one responding to traditional therapy and one requiring surgical management for drainage of a subperiosteal abscess.

## Case Report

This study was conducted in compliance with the Health Insurance Portability and Accountability Act and adhered to the tenants of the Declaration of Helsinki.

### Case 1

**Figure 1:**
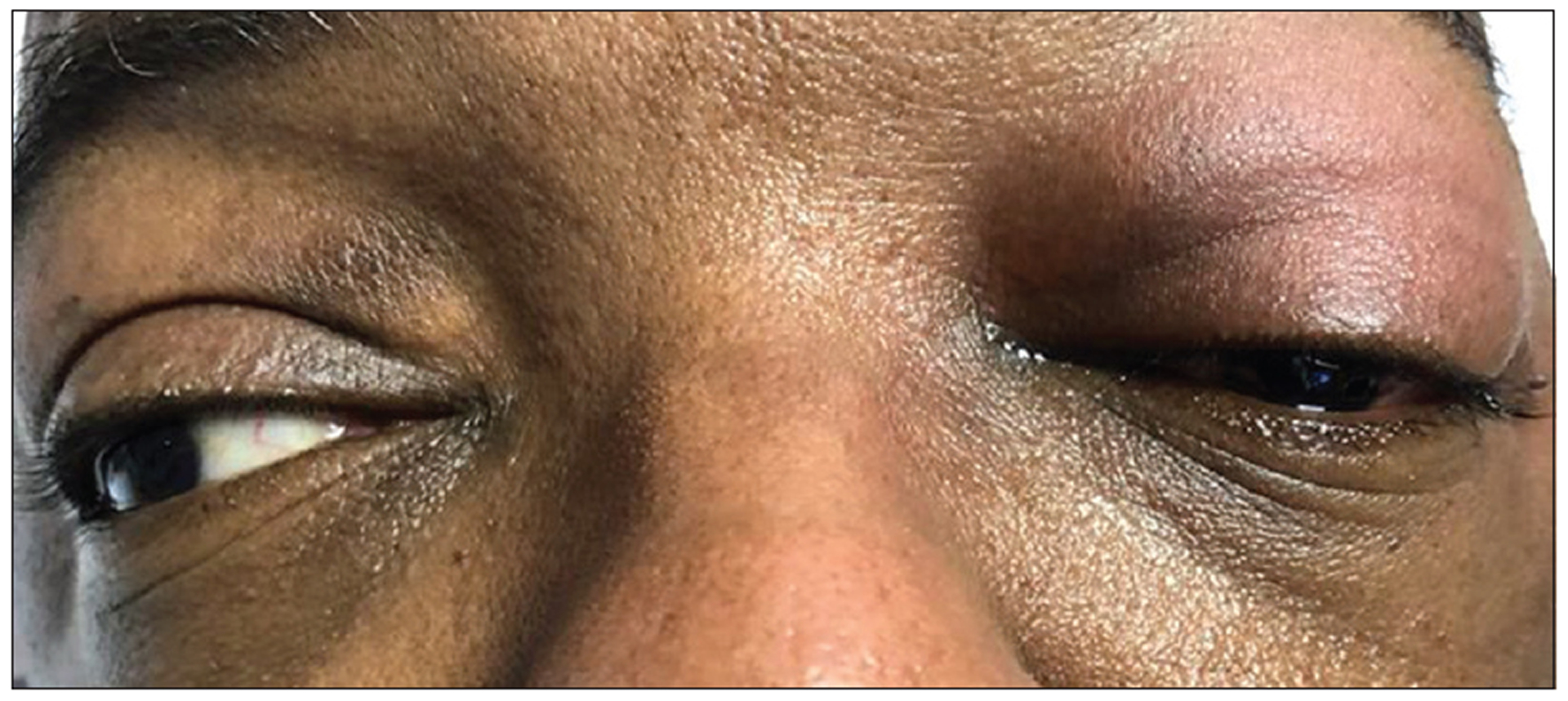
Clinical photograph of case 1 at the time of presentation

A 52-year-old male with a history of well controlled HIV on HAART (CD4 650) and nasal polyposis previously treated with FESS and currently on azelastine and Flonase presented with 2 weeks of progressive facial pain, congestion, and left periorbital swelling. He was treated as an outpatient with levofloxacin and steroids, but failed to improve. Examination revealed visual acuity of 20/20 OU, proptosis, and severe left upper eyelid edema and erythema [Fig. 1]. There was restriction of left extraocular movements in all fields of gaze and pain with abduction and supraduction. Rhinoscopy in the emergency department showed bilateral nasal cavities with copious secretions and polypoid tissue filling the middle meatus without necrotic eschars. He was afebrile with a normal white blood cell count and glucose.

**Figure 2:**
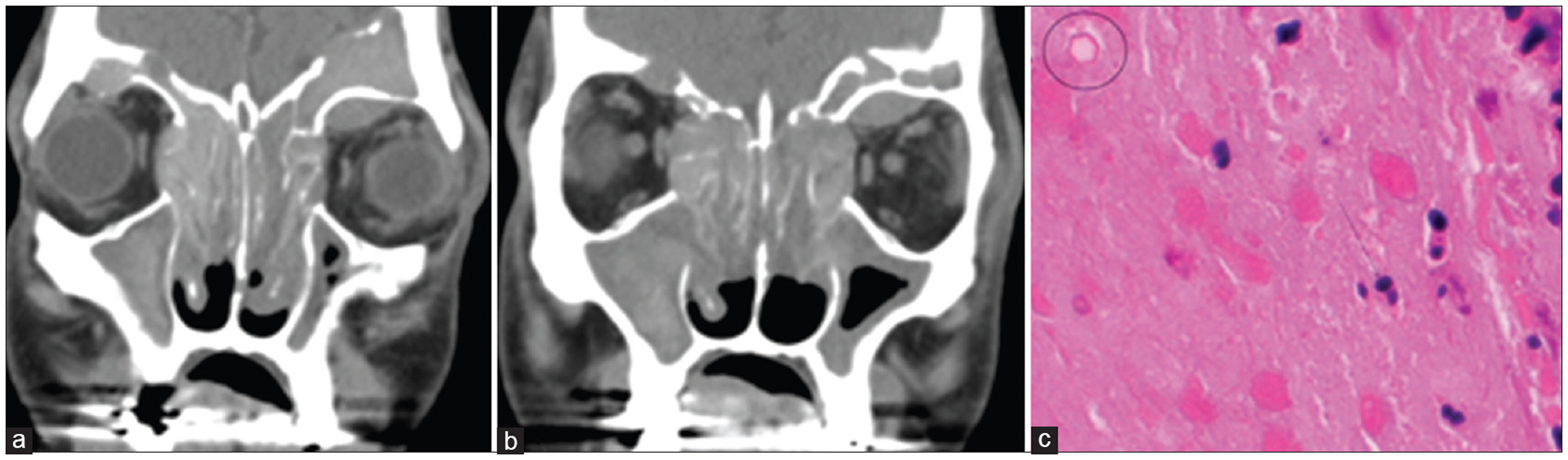
(a and b) CT Maxillofacial of patient 1 showing pan-rhinosinusitis with areas of osseous erosion and inflammatory changes extending into the orbital and intracranial compartments with a left intraorbital collection along the orbital roof. (c) Sinus biopsy showing eosinophils (arrow) and Charcot-Leyden Crystals (circle)

CT showed pansinusitis with areas of osseous erosion with inflammatory changes extending into the orbital and intracranial compartments with an intraorbital collection or infiltrate [Fig. 2a and b]. The patient’s history, immunocompetent status, physical examination, and imaging findings were most consistent with acute exacerbation of EMCRS with possible bacterial superinfection rather than invasive fungal sinusitis. The patient was started on prednisone and ampicillin/sulbactam which significantly improved his symptoms and exam findings. He subsequently underwent bilateral FESS. Pathology of the sinus mucosa showed allergic mucin with eosinophilia, benign bone with reactive changes, and no fungal organisms, consistent with EMCRS [Fig. 2c]. He was discharged on Augmentin and a prednisone taper. Examination 2 weeks post operatively showed full motility and complete resolution of periorbital edema. At one year follow-up, he was doing well and maintained on dupilumab and budesonide nasal rinses.

### Case 2

**Figure 3:**
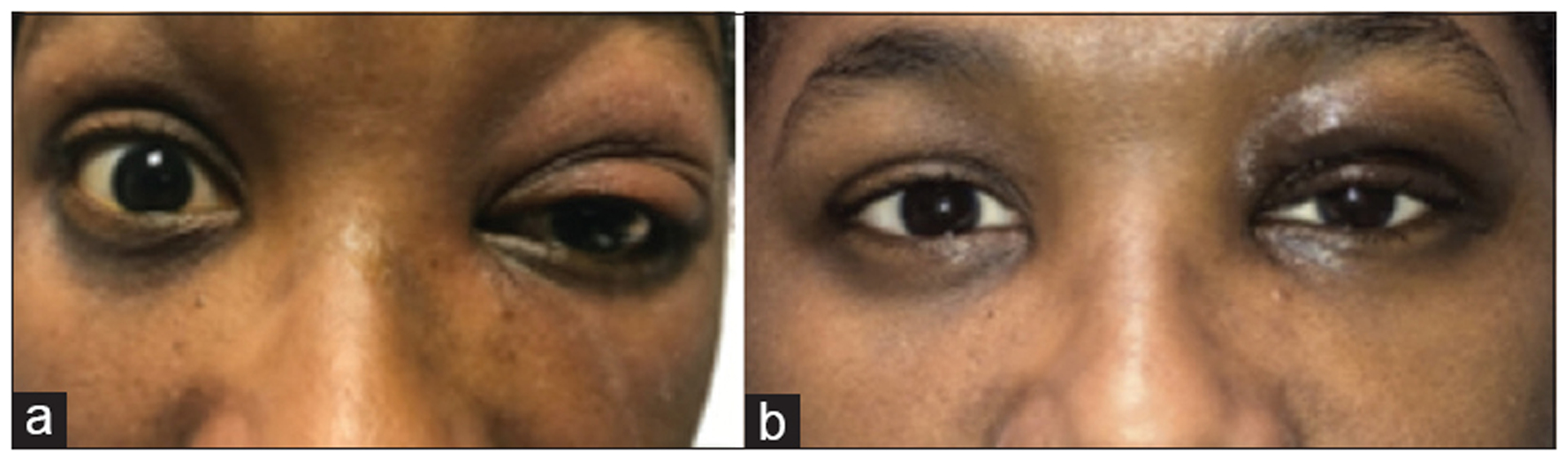
(a) Clinical exam on initial presentation showing left periorbital edema and mechanical left ptosis. (b) Clinical photo one week after drainage of subperiosteal abscess

**Figure 4:**
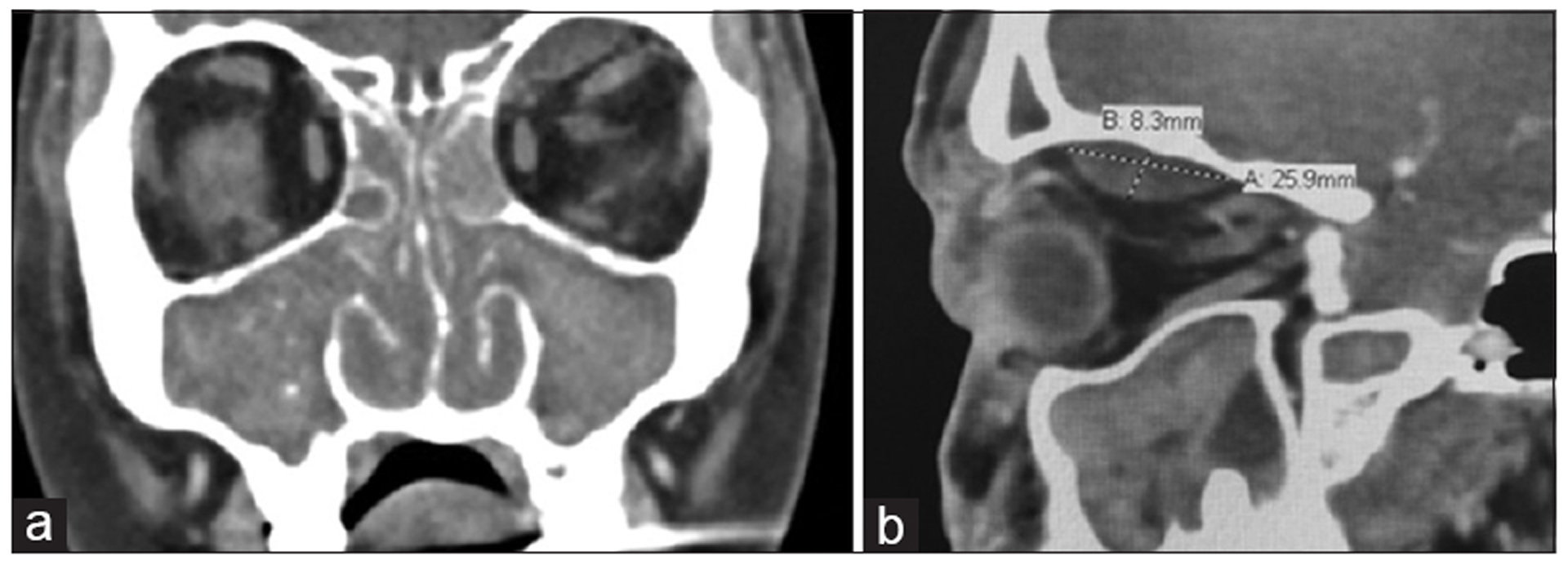
CT Face (a) coronal and (b) sagittal view of patient 2 showing opacification of the frontal, ethmoid, maxillary, and sphenoid sinuses with scattered areas of hyperdensity and a superior subperiosteal abscess without bony erosion measuring 8.3 mm × 25.9 mm

A 47-year-old female with a history of chronic sinusitis presented with six days of left sided headache, periorbital swelling, and pain with extraocular movements. Examination revealed visual acuity of 20/20 OU, mild left periorbital edema, and mechanical left ptosis [Fig. 3a]. Nasal endoscopy showed bilateral edematous mucosa with polyps without necrosis. CT showed opacification of the frontal, ethmoid, maxillary, and sphenoid sinuses, and findings suspicious for a superior subperiosteal abscess; there was no bony erosion between the sinus and orbit [Fig. 4]. Due to the absence of bone erosion but otherwise typical imaging features of EMCRS, there was concern for a superimposed bacterial infection causing a subperiosteal abscess. She was started on intravenous ceftriaxone, vancomycin, metronidazole, and dexamethasone. Relevant laboratory work up included negative HIV, normal hemoglobin A1C, normal white blood cell count, and negative aspergillus galactomannan. She subsequently underwent orbitotomy with incision and drainage of the subperiosteal abscess. An intraoperative microbial culture was unable to be obtained due to paucity of sample. Her periorbital edema and ptosis improved post-operatively [Fig. 3b]. She was discharged on a steroid taper. She subsequently underwent endoscopic sinus surgery with the otolaryngology service. Intraoperative pathology of the sinuses showed chronic inflammation and polypoid changes consistent with EMCRS. At 10 month follow-up, her chronic rhinosinusitis is controlled on budesonide irrigation.

## Discussion

AFS and EMCRS are thought to be related to impaired nasal mucosa causing stasis of secretions, chronic edema, and bacterial super infection.^[6]^ The aforementioned Bent and Kuhn criteria are widely accepted to diagnose AFS. The disease is most common in hot and humid climates.^[7]^ EMCRS has the same clinical and radiographic findings, but without evidence of fungus on histology or evidence of type I hypersensitivity.

Most patients with AFS have radiographic evidence of sinusitis involving multiple sinuses, most commonly ethmoid and maxillary. Bony erosion is also seen radiographically and is believed to be due to the pressure effect leading to bony remodeling of the surrounding facial skeleton, orbit, and skull base.^[5]^ Bone erosion with intracranial and/or intraorbital extension is much more common in AFS and EMCRS compared with other types of sinusitis.^[8]^

Effective treatment for AFS and EMCRS with ophthalmic manifestations includes systemic corticosteroids and endoscopic sinus surgery followed by medical management to prevent long-term recurrence.^[9,10]^ Anatomical orbital changes and bony remodeling may be reversed after adequate treatment.^[11]^ While periorbital abscesses secondary to bacterial sinusitis in adults are often surgically drained, this is usually not indicated for orbital collections seen in AFS and EMCRS, which extend from the sinuses into the orbit by bony erosion, as in our first case. Large sinusotomies with complete removal of the mucin is generally enough to manage the orbital involvement in such cases. However, the distinction between an intraorbital collection from AFS/EMCRS and a bacterial subperiosteal abscess should be made. There should be a higher degree of suspicion for a superimposed abscess when the fluid collection is not in continuity with the sinus through bony erosion. Meyer *et al*. reported such a unique case of AFS with orbital involvement associated with a large subperiosteal abscess necessitating anterior orbitotomy with drainage in the same setting as FESS.^[12]^

In both cases, patients were pretreated with antibiotics and oral steroids prior to sinus surgery. The clinical and radiographic presentation strongly suggested AFS/EMCRS rather than a neoplasm or an invasive fungal process. Pretreating with steroids significantly improves the acute inflammatory state leading to decreased bleeding and better surgical visualization. In the second case, given both the distortion of the anatomy from the compressive mucin as well as the surgical goal of complete removal of all mucin and polyps, the periosteal abscess was drained urgently and sinus surgery was staged at a later date. Our second case is unique in that there was no direct orbital extension of sinus disease by way of bone erosion.

## Conclusion

A low threshold for diagnosis of bacterial superinfection of the sequestered fluid led to a decision for orbital surgery in this particular case. Additionally, other life-threatening diagnoses such as malignancy must not be overlooked and should remain on the differential in any unusual case. Biopsies of nasal mucosa should be obtained during surgical intervention for presumed AFS/EMCRS to confirm the diagnosis. We highlight that multidisciplinary management of this sino-orbital disease by both otolaryngology and oculoplastics leads to early diagnosis and optimal treatment, especially in more challenging cases.
